# Changes in depression medication following the initial assessment by specialised psychiatry services in the Helsinki-Uusimaa Region

**DOI:** 10.1192/j.eurpsy.2025.681

**Published:** 2025-08-26

**Authors:** J. Juntura, P. Näätänen, G. Joffe, J. Ekelund, R.-L. Leskelä, T. Ito, B. Rive, Y. Godinov, I. Eriksson, P. Torkki

**Affiliations:** 1 University of Helsinki; 2 Department of Psychiatry, Helsinki University Hospital, Helsinki; 3 Nordic Healthcare Group, Espoo, Finland; 4 Janssen EMEA, High Wycombe, United Kingdom; 5 Janssen EMEA, Paris, France; 6 Janssen EMEA, Sofia, Bulgaria; 7 Janssen EMEA, Solna, Sweden

## Abstract

**Introduction:**

Depressive disorders often require specialised psychiatric services. Timely, appropriate medication initiation and/or change plays a crucial role in improving patient (pt) outcomes (Kraus *et al.* Transl Psychiatry 2019;9 127).

**Objectives:**

Describe the type of, and time to, medication changes within 12 months of the initial assessment of pts with depression recorded by specialised psychiatric care (SPC).

**Methods:**

This cohort study leveraged Finnish pt data from 19 registries from 2014–2020. Adult pts with a depression diagnosis recorded by SPC in the Helsinki and Uusimaa region in 2015 (with no depression diagnosis given by SPC within the previous year) were included. All treatments were recorded as monotherapy or combination/augmentation therapy. The Kaplan-Meier method was used to analyse time to treatment change (TTC).

**Results:**

9305 pts were included; baseline characteristics are reported (**Table 1**). There was no change to the baseline treatment status in 39.7% of pts (**Table 2**). The most common change was from no medication to monotherapy (2138 pts [45.6% of those with no treatment before]). 2202 (23.7%) pts remained untreated throughout the study. Median (95% confidence interval) TTC following the initial assessment by SPC was 53 (50–56) days (**Figure 1**).

**Image 1:**

**Figure:**
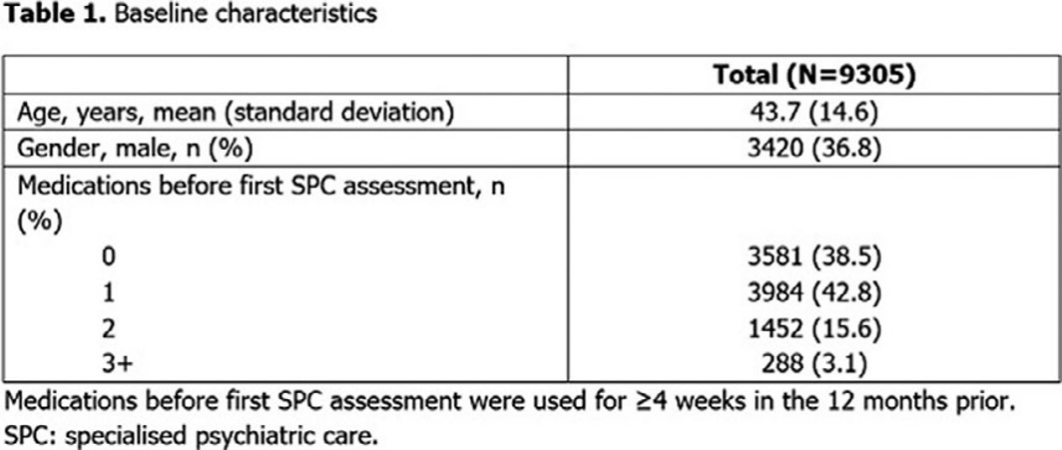

**Image 2:**

**Figure:**
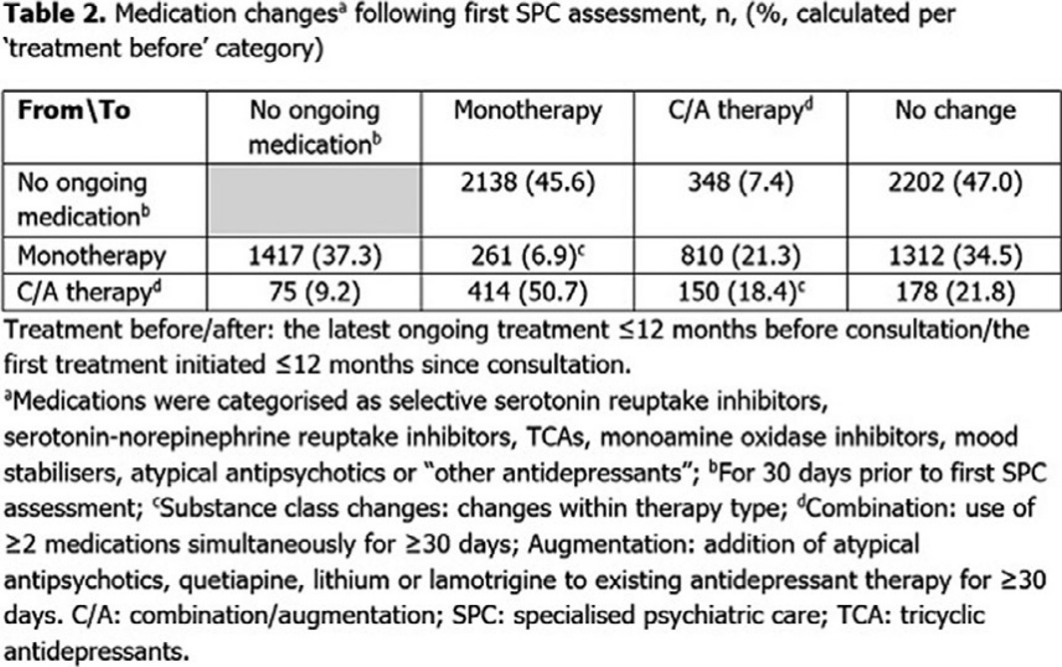

**Image 3:**

**Figure:**
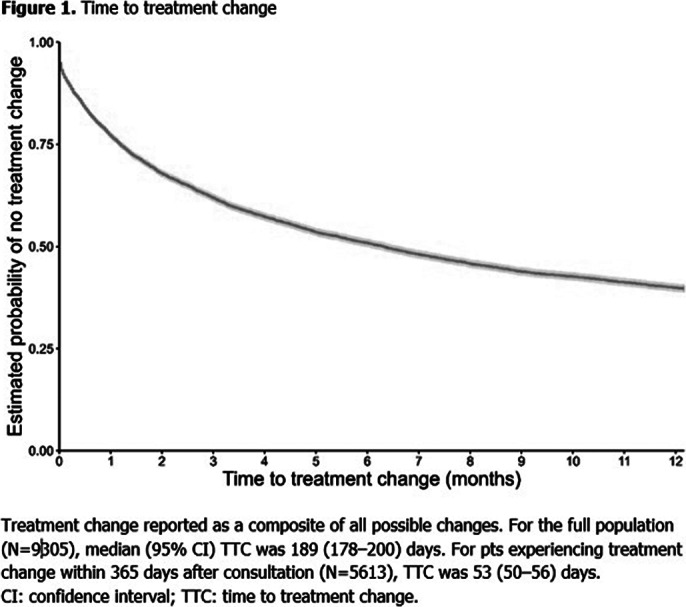

**Conclusions:**

Around 40% of pts referred to SPC had no prior pharmacotherapy. Monotherapy was the most common treatment provided. Almost 40% of pts had no change in their baseline treatment over 12 months, highlighting the need for further research to optimise care.

**Disclosure of Interest:**

None Declared

